# Left bundle branch area pacing in congenital heart disease

**DOI:** 10.1093/europace/euac175

**Published:** 2022-11-11

**Authors:** Matthew O’Connor, Omar Riad, Rui Shi, Dan Hunnybun, Wei Li, Julian W E Jarman, John Foran, Christopher A Rinaldi, Vias Markides, Michael A Gatzoulis, Tom Wong

**Affiliations:** Heart Rhythm Centre, Royal Brompton and Harefield Hospitals, Guy’s and St Thomas’ NHS Foundation Trust, London SW3 6NP, UK; Adult Congenital Heart Centre, Royal Brompton and Harefield Hospitals, Guy’s and St Thomas’ NHS Foundation Trust, Sydney Street, London SW3 6NP, UK; Department of Electrophysiology, Auckland City Hospital, Auckland 1023, New Zealand; Heart Rhythm Centre, Royal Brompton and Harefield Hospitals, Guy’s and St Thomas’ NHS Foundation Trust, London SW3 6NP, UK; Cardiology Department, Ain Shams University, Cairo 1181, Egypt; Heart Rhythm Centre, Royal Brompton and Harefield Hospitals, Guy’s and St Thomas’ NHS Foundation Trust, London SW3 6NP, UK; Heart Rhythm Centre, Royal Brompton and Harefield Hospitals, Guy’s and St Thomas’ NHS Foundation Trust, London SW3 6NP, UK; Heart Rhythm Centre, Royal Brompton and Harefield Hospitals, Guy’s and St Thomas’ NHS Foundation Trust, London SW3 6NP, UK; Adult Congenital Heart Centre, Royal Brompton and Harefield Hospitals, Guy’s and St Thomas’ NHS Foundation Trust, Sydney Street, London SW3 6NP, UK; Heart Rhythm Centre, Royal Brompton and Harefield Hospitals, Guy’s and St Thomas’ NHS Foundation Trust, London SW3 6NP, UK; Adult Congenital Heart Centre, Royal Brompton and Harefield Hospitals, Guy’s and St Thomas’ NHS Foundation Trust, Sydney Street, London SW3 6NP, UK; Heart Rhythm Centre, Royal Brompton and Harefield Hospitals, Guy’s and St Thomas’ NHS Foundation Trust, London SW3 6NP, UK; Kings College Hospital, London WC2R 2LS, UK; Heart Rhythm Centre, Royal Brompton and Harefield Hospitals, Guy’s and St Thomas’ NHS Foundation Trust, London SW3 6NP, UK; Adult Congenital Heart Centre, Royal Brompton and Harefield Hospitals, Guy’s and St Thomas’ NHS Foundation Trust, Sydney Street, London SW3 6NP, UK; National Heart & Lung Institute, Imperial College, London SW3 6LY, UK; Heart Rhythm Centre, Royal Brompton and Harefield Hospitals, Guy’s and St Thomas’ NHS Foundation Trust, London SW3 6NP, UK; Adult Congenital Heart Centre, Royal Brompton and Harefield Hospitals, Guy’s and St Thomas’ NHS Foundation Trust, Sydney Street, London SW3 6NP, UK; Kings College Hospital, London WC2R 2LS, UK; National Heart & Lung Institute, Imperial College, London SW3 6LY, UK

**Keywords:** Congenital heart disease, Conduction system pacing, Left bundle area pacing, Left bundle pacing, Bradycardia pacing

## Abstract

**Aims:**

Left bundle branch area pacing (LBBAP) has been shown to be effective and safe. Limited data are available on LBBAP in the congenital heart disease (CHD) population. This study aims to describe the feasibility and safety of LBBAP in CHD patients compared with non-CHD patients.

**Methods and results:**

This is a single-centre, non-randomized observational study recruiting consecutive patients with bradycardia indication. Demographic data, ECGs, imaging, and procedural data including lead parameters were recorded. A total of 39 patients were included: CHD group (*n* = 13) and non-CHD group (*n* = 26). Congenital heart disease patients were younger (55 ± 14.5 years vs. 73.2 ± 13.1, *P* < 0.001). Acute success was achieved in all CHD patients and 96% (25/26) of non-CHD patients. No complications were encountered in either group. The procedural time for CHD patients was comparable (96.4 ± 54 vs. 82.1 ± 37.9 min, *P* = 0.356). Sheath reshaping was required in 7 of 13 CHD patients but only in 1 of 26 non-CHD patients, reflecting the complex and distorted anatomy of the patients in this group. Lead parameters were similar in both groups; R wave (11 ± 7 mV vs. 11.5 ± 7.5, *P* = 0.881) and pacing threshold (0.6 ± 0.3 V vs. 0.7 ± 0.3, *P* = 0.392). Baseline QRS duration was longer in the CHD group (150 ± 28.2 vs. 118.6 ± 26.6 ms, *P* = 0.002). Despite a numerically greater reduction in QRS and a similar left ventricular activation time (65.9 ± 6.2 vs. 67 ± 16.8 ms, *P* = 0.840), the QRS remained longer in the CHD group (135.5 ± 22.4 vs. 106.9 ± 24.7 ms, *P* = 0.005).

**Conclusion:**

Left bundle branch area pacing is feasible and safe in CHD patients as compared to that in non-CHD patients. Procedural and fluoroscopy times did not differ between both groups. Lead parameters were satisfactory and stable over a short-term follow-up.

## Graphical Abstract

**Graphical Abstract:**
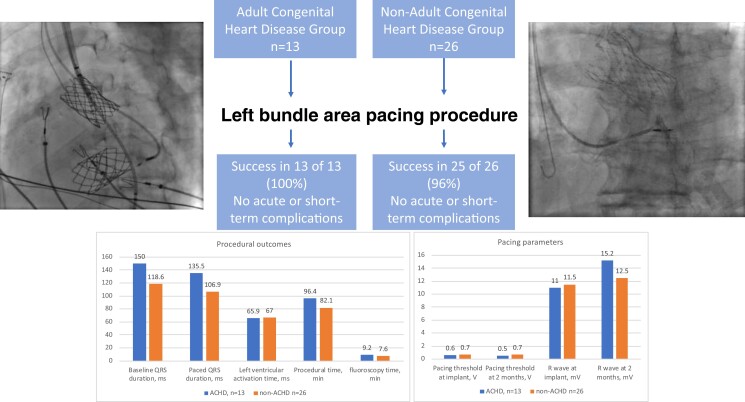

**What’s new?**

- Left bundle branch area pacing (LBBAP) is feasible and safe in the congenital heart disease (CHD) population.
- Procedural duration and electrical parameters are similar to those in non-CHD populations.
- The use of extensively and specifically reshaped sheaths is often required to achieve LBBAP in the CHD group.

## Introduction

Patients with congenital heart disease (CHD) are at a higher risk of developing a pacing requirement compared with the rest of the population due to both underlying conditions and surgical interventions.^1^ Furthermore, due to improved surgical and medical care, CHD patients are found to be living longer nowadays, and age-related conduction system disorders are now contributing to an increase in CHD pacing requirements.^2,3^ Traditional sub-pulmonary pacing is associated with the development of pacing-induced cardiomyopathy (PIC), heart failure, and increased mortality in both CHD and non-CHD patients.^4,5^ The risk of PIC increases with a pacing burden >20% and underlying ventricular dysfunction, the latter being a common finding in CHD.^6,7^

Pacing-induced cardiomyopathy can be mitigated (or treated) with cardiac resynchronization therapy (CRT), traditionally achieved with an additional epicardial lead implanted via the coronary sinus (CS). However, in CHD, this approach may be complex or not feasible due to associated anatomical lesions (dual ostial CS, unroofed CS) or prior surgery (CS re-routed to the pulmonary venous atrium). Conduction system pacing (CSP) has growing evidence of at least equivalence to traditional CRT.^8–12^ His bundle pacing (HBP) has been employed in CHD with good results,^12,13^ but evidence from non-CHD HBP studies has raised concerns about long-term threshold stability and poor sensing.^14^ Left bundle branch area pacing (LBBAP) overcomes these issues by implanting the lead distal to the His bundle, in the diffuse, arborized left bundle.^15^ Furthermore, the programming of LBBAP devices is less complex and this, in part, has facilitated its uptake across centres. Previous studies of CSP in CHD have been weighted towards HBP as LBBAP was still in its infancy but have highlighted this technique as a possible approach for CHD patients.^12,13^ In patients with CHD, the implantation of an LBBAP lead has additional challenges such as abnormal cardiac anatomy, for which the implant tools were not designed for, ventricular septal fibrosis or patch material and displaced conduction system tissue.

The aim of this study is to describe the technical considerations of LBBAP in CHD and to compare the feasibility, acute and short-term electrical and echocardiographic parameters of LBBAP in CHD as against a non-CHD population.

## Methods

### Study population

This is a prospective, non-randomized single-centre study including patients above 18 years who underwent elective LBBAP procedure mid-2020 to early 2022. Patients having left ventricular ejection fraction <50%, with bradycardia pacing indication, were also included for CRT. All patients provided informed consent, and the study was conducted in accordance with the declaration of Helsinki and was approved by the local ethics board. Demographic data including age, sex, body mass index (BMI), New York Heart Association (NYHA) class, past medical history of hypertension, diabetes, chronic kidney disease, and coronary artery disease was ascertained. Electrocardiogram data including rhythm, indication for pacing, QRS duration and imaging data including ventricular size and function, as well as septal late gadolinium enhancement (LGE) by cardiac magnetic resonance were collected.

### Left bundle pacing procedure

The LBBAP procedure was carried out under local anaesthetic or general anaesthetic if required by patient factors or the requirement for a subpectoral pocket. Venous access was obtained by either ultrasound-guided axillary vein access, venogram and fluoroscopy, or cephalic vein cut down. Procedural technique, definition of endpoints of LBBAP were previously described.^16^ Procedural data including QRS duration during LBBAP, LVAT, procedural and fluoroscopic times, radiation dose, intravenous contrast use were gathered. Lead parameters of the newly implanted LBBAP leads were carried out during the procedure via the programmer and included lead impedance, R wave, and pacing threshold data. A repeat check was performed within 24 h post-procedure.

### Follow-up

Patients were reviewed at 2 months to check symptoms, QRS duration on a 12 lead ECG, left ventricular function on an updated echocardiogram, and device programming.

### Statistical analysis

Continuous variables were expressed in mean ± SD, whereas categorical data were represented as frequency (percentage). Non-parametric data were described with median (interquartile range). Comparison between groups was performed using Student’s *t*-test and *χ*2 test. Data were analysed with Microsoft Excel and SPSS version 25 (SPSS, Chicago, IL, USA). Significant values are represented with a *P*-value <0.05.

## Results

### Baseline data

A total of 39 patients who had the LBBAP procedure were included and divided in a 1:2 fashion into two unmatched groups: CHD group (*n* = 13) and non-CHD (*n* = 26). Congenital heart disease patients were younger with a mean age of 55 ± 14.5 years compared with the non-CHD group, 73.2 ± 13.1 years (*P* < 0.001), while the female sex (46 vs. 42%, *P* = 0.819) and BMI (26 vs. 26, *P* = 0.914) were comparable between the two groups. The baseline mean QRS duration was longer in CHD group (150 ± 28.2 vs. 118.6 ± 26.6 ms, *P* = 0.002). The complete atrioventricular block was the commonest pacing indication in both groups, accounting for 54% in the CHD group and 73% in the non-CHD, whereas sinus node dysfunction was the indication in 38% of the CHD group (the latter had ventricular leads indicated due to extreme PR prolongation or high probability of future ventricular pacing support requirement). The pacing was required after cardiac surgery, during the same admission, in about one-third of both groups. Cardiovascular risk factors, coronary artery disease, and atrial fibrillation at baseline were more common in the non-CHD group (*Table 1*).

**Table 1:** Demographic and baseline data

	ACHD group (*n* = 13)	Non-ACHD group (*n* = 26)	*P*-value
Age (years)	55 ± 14.5	73.2 ± 13.1	<0.001*
Sex (women)	6 (46)	11 (42)	0.819
Body mass index (kg/m2)	26 ± 5.7	25.8 ± 4.0	0.914
Pacing indication			
ȃComplete AV block	7 (54)	19 (73)	
ȃSinus node dysfunction	5 (38)	0	
ȃPace and ablate AV node	1 (8)	6 (23)	
ȃHeart failure (CRT)	0	1 (4)	
Same admission post-cardiac surgery or TAVR	4 (31)	10 (38)	
Pre-existing device	0	2 (8)	
NYHA	2 ± 0.8	2 ± 0.7	0.858
Hypertension	1 (8)	9 (35)	
Diabetes mellitus	0	3 (12)	
Chronic kidney disease	0	2 (8)	
Coronary artery disease	0	3 (12)	
Baseline rhythm			
ȃSinus rhythm	11 (85)	15 (58)	0.093
ȃAtrial fibrillation	2 (15)	11 (42)	
LV ejection fraction	56 ± 8.2	52.8 ± 12.5	0.431
LV EDVI (mL/m2)	63 ± 13.3	49.6 ± 16.8	0.048*
LV ESVI (mL/m2)	28.6 ± 10.5	22.5 ± 11.2	0.181
Septal LV thickness (cm)	1 ± 0.2	1.1 ± 0.1	0.433
RV basal diameter (cm)	4.2 ± 1.1	3.8 ± 0.8	0.366
RV function			
ȃNormal	9 (70)	19 (73)	
ȃMildly reduced	2 (15)	7 (27)	
ȃModerately reduced	2 (15)	0	
Mitral regurgitation			
ȃNone/trivial	4 (32)	8 (31)	
ȃMild	5 (38)	9 (35)	
ȃModerate	2 (15)	2 (8)	
ȃSevere	2 (15)	1 (4)	
Tricuspid regurgitation			
ȃNone/trivial	4 (32)	7 (27)	
ȃMild	6 (45)	9 (35)	
ȃModerate	3 (23)	4 (15)	
ȃSevere	0	0	
LA volume index (mL/m2)	39 ± 23.2	37.9 ± 15.4	0.887
RA volume index (mL/m2)	37.9 ± 16.1	29.2 ± 17.1	0.268
Cardiac magnetic resonance scan performed	9 (69)	5 (19)	
Septal late gadolinium enhancement	9 (69)	2 (8)	

Continuous variables are expressed as mean ± SD, whereas categorical variables are expressed as numbers (percentage).

LV measurements are inclusive of measurements of the sub-pulmonary LV in the two TGA patients.

CRT, cardiac resynchronization therapy; EDVI, end-diastolic volume index; ESVI, end-systolic volume index; NYHA, New York Heart Association class; TAVR, transcutaneous aortic valve replacement.

*Statistically significant, *P* < 0.05.

### Congenital heart disease group anatomy was divided into three subgroups

- Simple: repaired atrioventricular or ventricular septal defects (*n* = 2), partial anomalous pulmonary venous drainage (*n* = 1), bicuspid aortic valve (*n* = 3), Shone’s syndrome (*n* = 1) and dextrocardia (*n* = 1).
- Moderate: repaired tetralogy of Fallot (rToF) (*n* = 3).
- Complex: transposition of great arteries (TGAs) with atrial-switch (Mustard) repair (*n* = 2).

The non-CHD group consisted of several subgroups: structurally normal heart, post-aortic and mitral interventions, post-transcutaneous aortic valve replacement, and dilated cardiomyopathy.

### Imaging

Mean LV ejection fraction, end-systolic LV volume, RV basal diameter, and left and right atrial volumes were similar between both groups, although LV end-diastolic volume was marginally larger in the CHD group (63 ± 13.3 vs. 49.6 ± 16.8, *P* = 0.048) (*Table 1*). Both patients with post-Mustard TGA had mildly dilated systemic right ventricle with mild to moderate reduction in systolic function. Moderate or severe mitral regurgitation was noted in four (30%) CHD patients and three (12%) in the non-CHD group, whereas moderate tricuspid regurgitation was present in three (23%) and four (15%) respectively. More cardiac magnetic resonance scans were performed in CHD patients (69 vs. 19%), in all CHD patients septal LGE was seen. In the non-CHD group, only two of five patients with MRI scans demonstrated septal LGE.

### Procedural outcomes

Left bundle branch area pacing was achieved in all CHD patients and 25 of 26 (96%) in the non-CHD group. In this case, deep septal tunnelling and thus LBBAP could not be achieved despite multiple attempts, and an RV septal lead was placed. Mean paced QRS duration was longer in the CHD group (135.5 ± 22.4 vs. 106.9 ± 24.7 ms, *P* = 0.005), with comparable reduction in QRS duration (19.3 ± 22.7 vs. 15.3 ± 25.2 ms, *P* = 0.681). Left ventricular activation time during LBBAP was similar between the CHD and non-CHD groups (65.9 ± 6.2 vs. 67 ± 16.8 ms, *P* = 0.840). The mean procedural time and fluoroscopy time were comparable between the CHD and non-CHD groups (96.4 ± 54 min vs. 82.1 ± 37.9 ms, *P* = 0.356 and 9.2 ± 4.2 vs. 7.6 ± 4.5 min, *P* = 0.312, respectively). There was no difference in mean radiation dose (1338.3 ± 947.7 vs. 1471.5 ± 1678.6 mGy·cm^2^, *P* = 0.808) or intravenous contrast volume (33.8 ± 16.4 vs. 26.1 ± 15.5 mL, *P* = 0.373) between the CHD and non-CHD groups, respectively. No acute complications occurred in either group. The median hospital stay was 1 day (range: 0–15) in the CHD group and 2.5 days (range 1–11.75) in the non-CHD group. Dual chamber pacemakers were the commonest devices in both groups, 11 of 13 patients (85%) in CHD and 73% in the non-CHD. Procedural and electrical data are depicted in *Table 2*.

**Table 2:** Procedural and electrical data

	ACHD group (*n* = 13)	Non-ACHD group (*n* = 26)	*P*-value
Procedural time (min)	96.4 ± 54	82.1 ± 37.9	0.356
Fluoroscopy time (min)	9.2 ± 4.2	7.6 ± 4.5	0.312
Radiation dose (mGy·cm^2^)	1338.3 ± 947.7	1471.5 ± 1678.6	0.808
IV contrast volume (mL)	33.8 ± 16.4	26.1 ± 15.5	0.373
QRS duration (ms)			
ȃBaseline	150 ± 28.2	118.6 ± 26.6	0.002*
ȃLeft bundle pacing (selective)	135.5 ± 22.4	106.9 ± 24.7	0.005*
Reduction in QRS duration (ms)	19.3 ± 22.7	15.3 ± 25.2	0.681
LVAT during LBBAP (ms)	65.9 ± 6.2	67 ± 16.8	0.840
Acute success	13 (100)	25 (96)	0.474
Acute complications	0	0	
Median hospital stay (days)	1 (0–15)	2.5 (1–11.75)	0.378
Venous access			
ȃAxillary	12 (92)	22 (85)	
ȃCephalic	1 (8)	4 (15)	
Generator location			
ȃSubcutaneous	13 (100)	25 (96)	
ȃSubpectoral	0	1 (4)	
Device type			
ȃSingle chamber	1 (7.7)	6 (23)	
ȃDual chamber	11 (85)	19 (73)	
ȃCRTD	1 (7.7)	1 (4)	

Continuous variables are expressed as mean ± SD, whereas categorical variables are expressed as numbers (percentage).

CRTD, cardiac resynchronization therapy defibrillator; LBBAP, left bundle branch area pacing; LVAT, left ventricular activation time.

*Statistically significant, *P* < 0.05.

### Pacing data

Lead parameters were similar between both CHD and non-CHD groups at implantation; R wave (11 ± 7 vs. 11.5 ± 7.5 mV, *P* = 0.881), pacing threshold at 0.4 ms pulse width (0.6 ± 0.3 vs. 0.7 ± 0.3 V, *P* = 0.392), and lead impedance (721.1 ± 289.3 vs. 682.5 ± 136.8Ω, *P* = 0.593). There was no significant change in lead parameters in either group in the 24 h check of the device.

### Description of implant

All device implants were performed using either the Medtronic (Minneapolis, MN, USA) C315H sheath and 3830 SelectSecure lead or the Boston Scientific (Marlborough, MA, USA) SSP3 sheath and Ingevity+ lead. A variety of advanced techniques were used in addition to the standard approach to LBBAP lead implantation. For patients with very dilated sub-pulmonary atria or ventricles, the C315H sheath did not easily find the appropriate pre-tunnelling position (determined by appropriate pace-map and fluoroscopic appearance) initially and two advanced techniques were employed:

- For patients where the predominant issue was a dilated RV, the C315H sheath was manually reshaped by increasing the primary curve giving it greater reach into the RV to reach the mid-septal location.
- For patients with severely dilated atria additional support to the system was generated by placing the C315H sheath inside a multi-purpose CS guide sheath with the proximal 12 cm cut off (including the haemostatic hub) taking care to retain purchase of the guide at all times.

**Figure 1:**
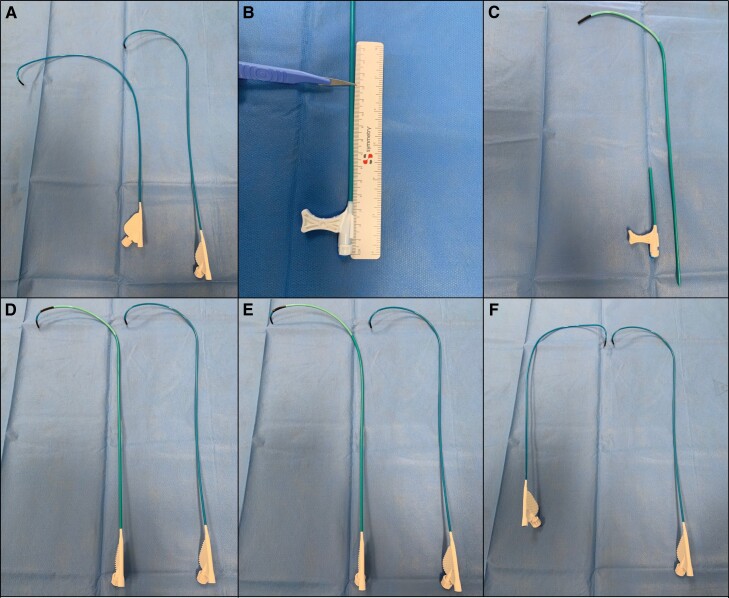
Sheath reshaping techniques for complex anatomy; all reshaping is performed with the dilator *in situ* to prevent sheath kinking or crush. (*A*) Exaggerated primary curve (left) for dilated RVs compared with ‘stock’ C315H. (*B* and *C*) Medtronic MPR CS guide with the proximal 12 cm cut off. (*D*) C315H sheath placed through a cut off MPR for extra sheath support (in cases of dilated RA). (*E*) Additional curve on MPR to address combined RA and RV dilation. (*F*) Complete reversal of the primary curve (left) for TGA with atrial-switch or dextrocardia anatomy.

For patients with significant chamber dilation and AV valve regurgitation, a combination of the above techniques was employed including manual reshaping of the CS guide to add a slight septal bias to the primary curve (*Figure 1*). In total, 7 of 13 cases required sheath reshaping compared with only 1/26 in the non-CHD group.

**Figure 2:**
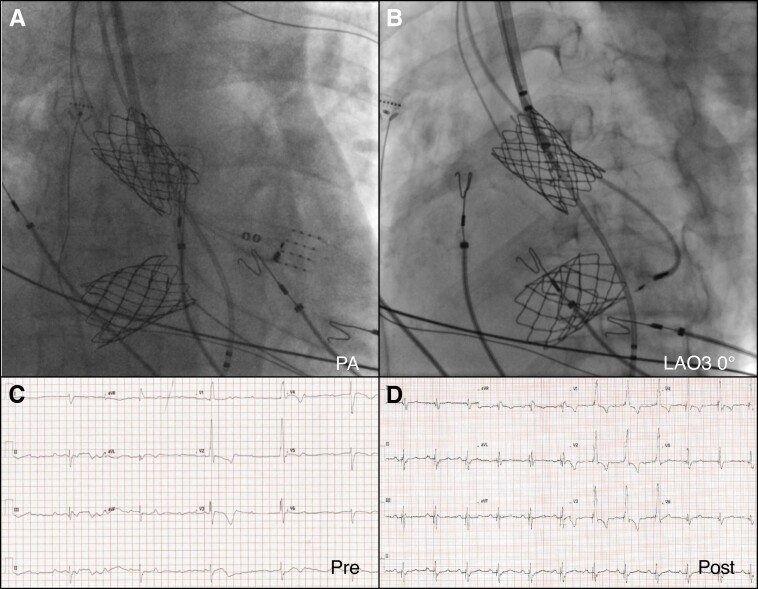
Left bundle branch area pacing in a patient with TGA with Mustard’s repair and stented systemic venous baffles. (*A*) fluoroscopic view in PA projection showing a multielectrode catheter (HD grid) for mapping of left bundle branch potentials with the 3830 SelectSecure lead within a (reshaped) C315H sheath and a quadripolar catheter for backup pacing in the sub-pulmonary left ventricle. (*B*) LAO projection at 30° showing contrast injection during lead tunnelling. (*C*) Baseline ECG showing complete AV block. (*D*) Post-procedure paced ECG with QRS duration of 160 ms. LAO, left anterior oblique; PA, posteroanterior.

For both patients with atrial-switch (Mustard), repairs for TGA 3D-electroanatomical mapping (EnSite-X, Abbott, IL, USA and CARTO, Biosense Webster, CA, USA) were used to identify the left bundle conduction system electrograms to guide implantation of the lead (see Supplementary material online, *Video S1*). In the case of a sub-pulmonary LV such as TGA post-Mustard or congenitally corrected TGA (cc-TGA), the left bundle is superficially located and thus does not require deep septal tunnelling to achieve LBBAP. However, due to the posterior nature of the LV the C315H sheath needs to be extensively reshaped by reversing the primary curve which produces an anteriorly (septally) directed secondary curve (*Figure 1* and Supplementary material online, *Video S2*). In this instance, clock torque at the proximal end of the sheath (instead of the usual counter-clock torque) achieves septal apposition and stability. Finally, one has to account for the septal leaflet of the sub-pulmonary (morphologically mitral) AV valve which obstructs direct access to the septum and requires the sheath to be inserted much more distally and retracted as opposed to in the RV where the typical LBBAP implant location is between the septal and anterior leaflets of the tricuspid valve (*Figure 2*).

**Figure 3:**
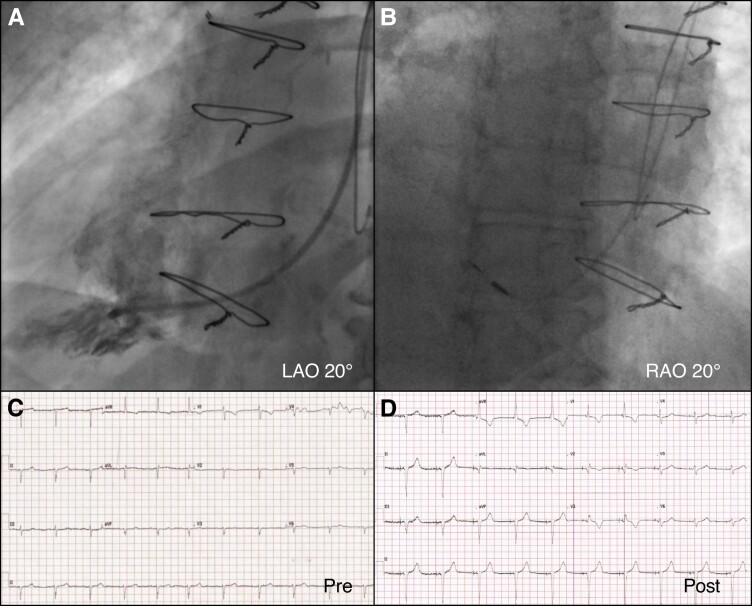
Left bundle area pacing in a patient with dextrocardia. (*A*) Right ventriculogram in LAO projection at 20° to identify anatomical landmarks. (*B*) Tunnelling process monitored in RAO 20°. The primary curve of the sheath was reversed to face the septum. (*C*) Baseline ECG showing sinus rhythm with negative QRS in V4-V6, I and aVL, typical of dextrocardia with QRS duration of 96 ms. Pacing indication was 6 s sinus pauses. (*D*) Post-procedural ECG showing QRS duration of 114 ms. LAO, left anterior oblique; RAO, right anterior oblique.

In the case of dextrocardia, a posterior secondary curve is still required, but due to the right-sided heart, the same primary curve reversal, as for the Mustard anatomy, is required to achieve the posterior angulation of the secondary curve. Once the primary curve has been reversed clock torque at the proximal end of the sheath continues to improve septal apposition and stability as in the TGA/Mustard configuration (*Figure 3*).

**Figure 4:**
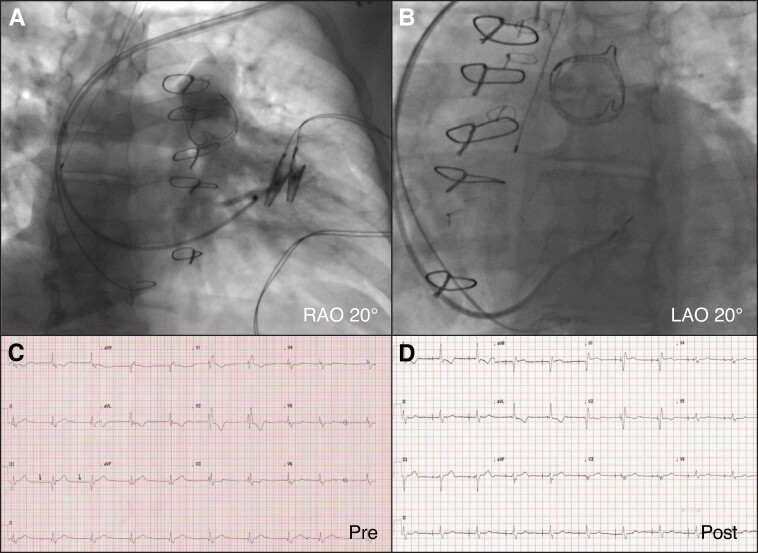
Left bundle area pacing in a patient with rToF and pulmonary valve prosthesis. (*A*) Right ventriculogram through the C315H sheath with augmented primary curve, in RAO projection at 20°, revealing the desired location for lead tunnelling, 2 cm distal to the expected His bundle location at the tricuspid valve plane. (*B*) Right ventriculogram in LAO projection at 20° showing the lead tip tunnelled through the septum, marked by the contrast edge. (*C*) Baseline ECG showing sinus rhythm (arrows indicate *P* waves), with first degree AV block and right bundle branch block, QRS duration 175 ms. (*D*) Post-procedural ECG during left bundle pacing with QRS duration of 148 ms. LAO, left anterior oblique; RAO, right anterior oblique.

For patients with rToF, the VSD patch necessitates a more apical implant to move beyond the patch material for tunnelling, this itself did not require extensive sheath reshaping, but two of three cases required additional reach with the primary being exaggerated. In our experience, the lead did not need to be tunnelled as deep to capture the left bundle (as per contrast septogram after lead deployment). Despite the, often extensive, septal scarring seen on invasive mapping there was no difficulty in achieving lead tunnelling in these patients (*Figure 4*).

### Follow-up

**Figure 5:**
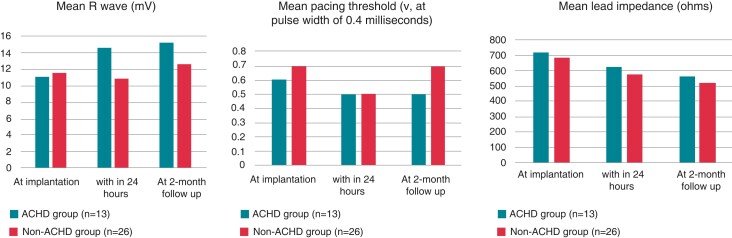
Pacing parameters at implantation, with 24 h post-procedure and at 2-month follow-up.

At 2-month follow-up, pacing threshold (at 0.4 ms pulse width) was lower in CHD group (0.5 ± 0.1 V vs. 0.7 ± 0.2, *P* = 0.026) while lead impedance and R waves were stable and comparable between both groups; lead impedance (562.4 ± 112.2 vs. 521.8 ± 81.6Ω, *P* = 0.263), R wave (15.2 ± 7.5 mV vs. 12.5 ± 7.0, *P* = 0.381), respectively. Pacing data are detailed in *Table 3* and *Figure 5*.

**Table 3:** Pacing data at implantation, within 24 h after implantation, and after 2 months

	ACHD group (*n* = 13)	Non-ACHD group (*n* = 26)	*P*-value
At implantation			
ȃLead impedance (Ω)	721.1 ± 289.3	682.5 ± 136.8	0.593
ȃR wave (mV)	11 ± 7	11.5 ± 7.5	0.881
ȃPacing threshold (V, pulse width 0.4 ms)	0.6 ± 0.3	0.7 ± 0.3	0.392
Post implantation (within 24 h)			
ȃLead impedance (Ω)	625.8 ± 175.8	574.6 ± 103.8	0.265
ȃR wave (mV)	14.6 ± 5.9	10.9 ± 6.6	0.168
ȃPacing threshold (V, pulse width 0.4 ms)	0.5 ± 0.2	0.5 ± 0.1	0.812
ȃAtrial pacing (%)	55.1 ± 37.1	8.4 ± 19.3	<0.001*
ȃVentricular pacing (%)	74.2 ± 42.4	76.8 ± 37.7	0.850
Post implantation (2-month follow-up)			
ȃLead impedance (Ω)	562.4 ± 112.2	521.8 ± 81.6	0.263
ȃR wave (mV)	15.2 ± 7.5	12.5 ± 7.0	0.381
ȃPacing threshold (V, pulse width 0.4 ms)	0.5 ± 0.1	0.7 ± 0.2	0.026*

Continuous variables are expressed as mean ± SD, whereas categorical variables are expressed as numbers (percentage).

*Statistically significant, *P* < 0.05.

## Discussion

The main finding in our series is that LBBAP for bradycardia indication is feasible and safe in patients with CHD with comparable success rates to non-CHD patients. Procedural and fluoroscopy times were also similar. Two-month follow-up showed stable pacing parameters in both groups.

Few studies have evaluated LBBAP outcomes in the CHD population, mainly as part of CSP, with a majority of cases having HBP.^12,13,17^ Some case reports have also described LBBAP in CHD.^18,19^

Left bundle branch area pacing in the CHD population requires expertise in CSP, and knowledge of congenital heart defects and subsequent corrective surgeries. Pre-procedural imaging with CT or cardiac MRI enables the appreciation of the congenital defect, size and orientation of cardiac structures, and venous anatomy. Furthermore, prior knowledge of septal thickness and the location of the septal scar is helpful in procedural planning and guiding sheath reshaping and anticipating the required tunnelling depth.

Modification of the sheath curvature, to suit the underlying anatomy increases the chances of procedural success and was required in over 50% over cases in this cohort. This reflects the complex anatomy further highlighting the required expertise and knowledge in CHD for procedural success. Sheath reshaping ranged from minor, such as in the case of rToF where a slight increase in the primary curve was required, to substantial such as in the cases of TGA with Mustard repair or dextrocardia where complete primary curve reversal was required. The C315H sheath is amenable to reshaping and holds the modified shape well for a period of 5–10 min in our experience; after this duration, the sheath shape should be re-evaluated as it will have softened and partially straightened. Although extensive reshaping is possible, it is important it is performed with the dilator *in situ* to prevent sheath kinking which will impede lead rotations during deployment.

In addition to sheath reshaping, we utilized 3D mapping in the two cases of TGA with a Mustard repair; although the left bundle is superficially located in the sub-pulmonary LV the additional tortuosity of superior access and potential for significant congenital and post-surgical cardiac rotation make LBBAP in this patient group complex and we find that demarcation of the proximal conduction system with 3D mapping to be invaluable. In addition to placing the lead with a mapping catheter positioned over the target area (*Figure 2*) as a fluoroscopic marker in both CARTO and EnSite-X, it is possible to visualize the lead tip so as to guide placement as a secondary confirmation of the position.

The decision to undertake LBBAP in the systemic RV patients was based on the previously demonstrated importance of sub-pulmonary LV function in this group. Single-site sub-pulmonary ventricular pacing has been shown to be an independent predictor of systemic RV dysfunction in cc-TGA.^5^ This risk of PIC is higher in patients with longer intrinsic or paced QRS durations, depressed baseline ventricular function, and those with a higher pacing burden.^6,20^ Synchronous sub-pulmonary LV activation (as provided by LBBAP) thus remains important as sub-pulmonary LV dysfunction is associated with clinical heart failure.^21^ Traditional bi-ventricular CRT has shown benefit with an increase in RV fractional area change from 18 to 30% in the systemic RV population, whether LBBAP has a similar effect is not known.^22^ Pacing the right bundle, in this situation, is conceptually attractive, but the anatomical nature of the RB, a thin, discrete, bundle would make targeting it very challenging. His bundle pacing (including right bundle capture) would potentially be superior in this situation, but this has to be balanced against the limitations and long-term safety concerns of HBP.

Despite achieving similar LVATs in both CHD and non-CHD groups the resultant QRS duration remained longer in the CHD group. Though this is potentially related to the longer QRS duration at baseline seen in CHD patients it is likely also to represent conduction delay due to fixed scar that cannot be overcome. Although LVAT and QRS duration are related, the LVAT is a better measure of left ventricular synchrony, and the QRS being influenced by the RV activation which is often slower in CHD patients due to RV dilation or prior surgical scar. The clinical relevance of a longer QRS duration despite similar LVATs is not known.

The short LVAT achieved in our study of 66 ms suggests true left bundle capture. Left bundle branch area pacing with left bundle capture pacing results in optimal left ventricular synchronization but may also result in greater interventricular dyssynchrony compared with left ventricular septal pacing.^23,24^ In the CHD population, the haemodynamic interplay of ventricular and interventricular synchronization is complex, and therefore, device programming should be individualized. With the goal of optimal cardiac resynchronization, it is possible that anodal capture and subsequent RV septal ‘pre-excitation’ would reduce interventricular dyssynchrony. Additionally, AV optimization to achieve fusion with intrinsic conduction (when possible) may improve overall ventricular synchrony.

In our study, acute success in LBBAP was achieved in all patients in the CHD group and 25 of 26 (96%) in the non-CHD group with no acute complications. Similarly, Moore *et al*.^12^ studied the outcomes of CSP in a cohort of 15 cc-TGA patients. HBP was performed in 13 of 15 (86%) and two of them had LBBAP, all with no complications. Cano *et al*.^13^ described acute success in CSP in 15 of 20 (75%) patients who had mainly complex and moderate congenital heart defects, of which five patients underwent LBBAP. One patient in the LBBAP group required lead revision for pacing threshold rise.

We found that the mean procedural and fluoroscopy time for LBBAP in the CHD group (96.4 ± 54 and 9.2 ± 4.2 min, respectively) did not significantly differ from that in the non-CHD group. In contrast, Cano *et al*. reported a mean procedural time of 148 ± 88 min for CSP in 15 patients, 5 of whom had LBBAP, whereas the remainder had HBP. Also, Moore *et al*. recorded procedural times of 146 min (Interquartile ragnge: 112–212) for CSP (mainly HBP, 86%) in 15 patients with cc-TGA. Our shorter procedural time could be due to the use of LBBAP rather than HBP, and fewer patients in our study were in the complex and moderate CHD categories compared with those in these studies.^12,13^

Lower pacing thresholds and better R wave sensing are one of the advantages of LBBAP. In our series, pacing threshold, R wave sensing, and lead impedance were satisfactory at the time of implantation and remained stable for a follow-up period of 2 months. Cano *et al*.^13^ also described favourable pacing parameters at implantation and at a median follow-up of 478 days (interquartile range: 225–567). Moore *et al*.^12^ showed stable lead parameters for 8 months in cc-TGA patients undergoing CSP.

The results of this study should be taken in the context of its observational nature and a relatively small, heterogenous cohort compared with unmatched control. The short-term follow-up in our study does not provide information about the long-term durability of leads in the deep septal location, although data in non-CHD patients show that lead parameters are stable out to 1 year.^25^ As is the case with all deep septal leads, the outcomes of lead extraction are unknown, and this should be taken into consideration, given the younger age of the CHD population.

## Conclusions

Left bundle branch area pacing for bradycardia indication in patients with CHD appears to be feasible and safe, with procedural and fluoroscopy times comparable to non-CHD patients. The pacing parameters became stable in both groups over a 2-month follow-up. Given the potential benefits of CSP in this population, LBBAP is a promising new pacing technique.

## Supplementary Material

euac175_Supplementary_Data

## Data Availability

All data are available upon reasonable request to the corresponding author.
